# Artificial Intelligence in higher education: use and appropriation of tools by university students

**DOI:** 10.3389/fpsyg.2026.1840886

**Published:** 2026-07-03

**Authors:** Raúl González, Roberto Valdés, Esteban Pérez, Ernesto Leon-Castro

**Affiliations:** 1 Department of Administrative Sciences, Universidad Autónoma del Estado de Baja California, Tijuana, Baja California, Mexico; 2 Faculty of Economics and Administrative Sciences, Universidad Católica de La Santísima Concepción, Concepcion, Chile

**Keywords:** academic performance, AI, higher education, learning, university students

## Abstract

**Introduction:**

This research presents the results of the study to analyze the perception of Artificial Intelligence (AI) and its relationship with the learning and Academic Performance of students at a university in northwestern Mexico during the period February to May 2025.

**Method:**

An analytical, cross-sectional study was conducted. The students in the study were selected through non-probabilistic sampling, comprising 289 students in the third, fourth, and sixth semesters. Those who did not complete the questionnaire correctly were excluded from this sampling. The instrument was developed using a Likert-type scale with responses: strongly disagree, disagree, indifferent, agree, and strongly agree.

**Results:**

The results of the present research confirm that integrating AI-based tools into educational environments is changing how students relate to knowledge, promoting more personalized and adaptive learning experiences. by means of confirmatory factor analysis (CFA), an analysis procedure in structural equation modeling (SEM).

## 1 Introduction

Higher education is undergoing a structural transformation driven by the expansion of digitalization, the intensification of globalization, and the rapid development of emerging technologies. These factors have significantly altered the institutional, pedagogical, and organizational dynamics of universities, generating new models of teaching, learning, and knowledge production. These changes have significantly influenced how students access information, interact with knowledge, and participate in academic learning environments (Bond et al., 2024). In this context, the widespread availability of digital tools has reshaped learning practices, enabling university students to access knowledge beyond traditional classroom settings and engage with more flexible and technology-mediated educational experiences (Zawacki-Richter et al., 2019). Among emerging technologies, artificial intelligence (AI) has become one of the most influential developments in contemporary higher education. Recent advances in generative AI systems have expanded the possibilities for supporting students' learning processes by providing adaptive feedback, automated explanations, and access to vast amounts of information in real time (Kasneci et al., 2023). Consequently, AI is increasingly integrated into students' academic practices, transforming how they conduct research, write assignments, and solve complex problems (Dwivedi et al., 2023). These technologies contribute to the development of more personalized learning environments, allowing students to receive tailored academic support based on their individual needs and learning patterns (Zawacki-Richter et al., 2019; Holmes et al., 2022).

To better understand the adoption and use of generative AI among university students, this study draws on two complementary theoretical frameworks: the SAMR model, which explains the levels of technology integration in educational practices, and the Unified Theory of Acceptance and Use of Technology (UTAUT), which analyzes the factors influencing users' acceptance and behavioral intention toward new technologies. These frameworks provide a structured perspective for examining how students incorporate AI tools into their academic workflows and how such tools transform traditional learning activities. AI-driven educational tools offer several potential benefits for university students. For instance, intelligent systems can analyze learning behaviors, provide immediate feedback, and help students identify gaps in their knowledge, thereby supporting more effective and autonomous learning processes (Holmes et al., 2022). Generative AI platforms, such as large language models and AI-assisted writing tools, can facilitate academic tasks including literature exploration, idea generation, and drafting of written assignments (Kasneci et al., 2023). Moreover, AI-supported systems can enhance students' engagement by enabling interactive learning experiences and offering personalized academic guidance (Luckin et al., 2022).

At the same time, AI applications may support research activities among university students by assisting in the organization of academic sources, identifying patterns within large datasets, and summarizing complex information (Dwivedi et al., 2023). These capabilities have the potential to improve students' efficiency in information processing and knowledge construction, particularly in disciplines that require intensive engagement with scientific literature and data analysis (Holmes et al., 2022). Despite these opportunities, the increasing reliance on AI tools among university students has also raised several academic, ethical, and pedagogical concerns. One of the most frequently discussed issues relates to academic integrity, as generative AI systems may produce content that students could submit as their own work without proper attribution (Cotton et al., 2024). Additionally, concerns have emerged regarding the accuracy and reliability of AI-generated outputs, since these systems can occasionally produce misleading or fabricated information (Kasneci et al., 2023). Another important challenge involves the potential impact of excessive reliance on AI technologies on students' cognitive development. Scholars have argued that overdependence on automated tools could weaken essential academic competencies such as critical thinking, problem-solving, and independent knowledge construction (Dwivedi et al., 2023). Furthermore, the use of AI in educational contexts raises important questions related to data privacy, algorithmic transparency, and the ethical management of students' personal information (Holmes et al., 2022). Addressing these concerns requires the development of clear institutional policies, responsible AI practices, and digital literacy initiatives that enable students to use AI tools in a critical and ethical manner.

## 2 Materials and methods

### 2.1 Theoretical framework

To understand the meaning of Artificial Intelligence, Rouhiainen (2018) defines AI as the ability of computers to perform activities that normally require human intelligence. This is because AI is the ability of machines to use algorithms, learn from data, and apply what they learn to decision-making, just as a human would. The development of Artificial Intelligence is divided into three stages, each with distinct capabilities. In the first stage, the software operates according to logical rules so that a system can act intelligently, such as playing chess. In the second stage of AI, machine learning techniques are used to extract patterns and knowledge from large amounts of data, which is where AI is currently. In the third stage, it is estimated that the basis of artificial superintelligence will be established, with systems capable of understanding the real world and offering innovative solutions to new or unknown problems (Franganillo, 2023).

According to Abeliuk and Gutiérrez (2021), in their research, they cite Lovelace, who in 1842 programmed the first algorithm to be processed by a machine. They also mention that machines could act on other things besides making mathematical calculations, such as composing elaborate and scientific musical pieces of any degree of complexity. Artificial Intelligence is already part of our lives: social networks, the applications we use on our phones, and other domestic devices have integrated it into the activities we carry out in our daily lives (López Baroni, 2019). Tech companies created AI for use in their businesses; for example, Google uses AI for its search engine, and Facebook uses it for targeted advertising and photo tagging. But the use of AI extends well beyond the technology sector. Drones and autonomous weapons use AI to kill without human intervention, and courts have implemented AI to make decisions. AI can now detect human faces not only to identify them, but also to interpret emotions and retrieve all kinds of information (Coeckelbergh, 2021).

Currently, Artificial Intelligence has been integrated into many fields, with its impact evident in medicine, finance, law, industry, entertainment, and education. Artificial Intelligence tools for education have experienced rapid growth worldwide and have impacted higher education institutions in Latin America (Aguilar et al., 2023). The authors Nogueira (2019) summarized the definitions of Artificial Intelligence in four cores: systems that think like human beings, systems that think rationally, systems that act like human beings, and systems that act rationally.

The term Artificial Intelligence was first introduced at a conference at Dartmouth University in 1956, and institutions such as UNESCO and ISO/IEC have since developed a glossary of basic terms to promote literacy in the use of Artificial Intelligence-related terms. Among the necessary terms that make up this glossary are: Algorithm, machine learning, deep learning, strong artificial intelligence, weak artificial intelligence, Big Data, and neural network (Peña et al., 2020; De Jesús Molina et al., 2025).

Algorithms: Briva-Iglesias (2023) defines algorithms as software that uses data through mathematical calculations to find repeating patterns and make predictions. Predictions allow certain decisions to be made and certain tasks that have traditionally been associated with human intelligence, such as image detection, to be performed. In this sense, Machine Learning, according to Díaz-Ramírez (2021), is the ability of computers to learn from data; other researchers mention that computers learn from experience. This type of AI has been growing quite rapidly due to the amount of data available today. With machine learning, large amounts of data are used, including many examples that produce the desired result. Through this process, the computer learns and builds a model with rules that generate new ideas or responses for the user (González-Videgaray and Romero-Ruiz, 2022).

Deep learning is a set of algorithms that use massive amounts of data from a specific domain to make decisions that optimize the desired outcome. To obtain results, algorithms train themselves to recognize patterns and correlations that are invisible or irrelevant to humans, and to connect data points to the desired outcome. Deep learning is used, for example, in lending, using relevant data about borrowers, such as credit score, income, and recent credit card usage, to minimize default rates. Deep learning is also used in autonomous vehicles to recognize patterns in camera pixels and use that information to plot the safest route (Lee, 2020).

Artificial Intelligence has different classifications; the most used or popular is Generative Artificial Intelligence, which refers to methods and applications capable of generating content, such as text and images, with characteristics indistinguishable from those produced by a human being (Corredera, 2023). Generative artificial intelligence has impacted the educational landscape by facilitating the creation of innovative and personalized content, promoting new forms of interaction between students and teachers, and acting not only as support tools but also as transformative elements within contemporary education (Vaerenbergh, 2024). The Director-General of UNESCO declared at Mobile Learning Week that Artificial Intelligence will profoundly transform education, with teaching methods changing as well as the way knowledge is acquired and information is accessed. Merging Artificial Intelligence with pedagogical knowledge, this will lead to the creation of learning environments that adapt to students' needs (Tafur and Molina, 2023).

### 2.2 AI tools for students

Ruiz (2023) presents a periodic table based on the taxonomy of Anderson and Krathwohl and the periodic table of Andrea Oviedo. In this periodic table, various Artificial Intelligence tools are represented, categorized according to the taxonomy in which they are located. The first scale is “Create”: tools that allow you to generate new ideas, products, or perspectives. The second scale, “Evaluate,” includes tools that allow information to be broken down into parts to understand its structure and relationships. The “Apply” scale refers to tools that use the information acquired in new situations. The “Understand” scale includes tools that help you interpret, compare, and contrast information. And finally, the “Remember” level includes tools capable of remembering or retrieving previously learned information.

ChatGPT is an example of a Generative Artificial Intelligence application that, through a prompt with a conventional chat format, allows the user to write textual instructions for the image or text to be generated. The technical foundations of Generative Artificial Intelligence are not new; generation is not done by innate intelligence; machines do not have innate intelligence; the machine “learns” through a massive training process, making decisions based on already existing and validated data. ChatGPT is one of the most popular and widely used artificial intelligence tools. Its name comes from the acronym Generative Pretrained Transformer. The company OpenAI presented this tool in November 2022, a conventional chatbot that generates text from users' questions, capable of creating “original” and highly accurate content. This tool reached 100 million users just 2 months after its launch. This type of Artificial Intelligence model is trained on large datasets to learn to predict the next word in a sentence and thus generate a coherent and convincing response to a question or instruction (Sabzalieva and Valentini, 2023). Another very relevant application of ChatGPT in the educational field is in mathematics and statistics, where the synergy between machine learning and computational simulation improves the accuracy and efficiency of predictive models (Dumancela et al., 2024).

The company OpenAI developed another generative artificial intelligence tool called DALL-E. This tool is configured as an artificial neural network for generating images from textual descriptions. This program creates high-quality images that are faithful to the user-provided textual instructions in a very fast way, imitating various styles and techniques, as well as creating hyperrealistic pieces (Añez Held, 2023). According to Sánchez (2023), this is achieved through a prompt, a textual instruction provided to the artificial intelligence model to guide it in generating a specific image, transforming the image's pixels over time to create the final image. ChatGPT was asked to describe what DALL-E is, and it responded as follows: “DALL-E is an artificial intelligence model developed by OpenAI that generates images from natural language descriptions. Its name combines “Dalí” (after the surrealist painter Salvador Dalí) and “WALL· E” (Pixar's robot), reflecting its creative and technological capacity. In addition to this answer, ChatGPT notes that DALL-E demonstrates creative generation, as it can combine unusual objects or ideas into coherent scenes. It also allows you to edit images by modifying parts of an existing image to add, remove, or alter elements, as well as to use techniques such as watercolor, oil, photorealism, and digital illustration, among others.

Another widely used tool for creating images with Generative Artificial Intelligence is Midjourney. According to the Company, “Midjourney” is an independent research laboratory that explores new means of thought and expands the imaginative powers of the human species (Campo-Ruiz, 2025). This tool is possible thanks to recent advances in hardware that increase computational power and the large amount of data generated over the past decades, which enable the training of generative AI and the creation of images at the level of highly competent photographers, illustrators, and artists (Corzo, 2025). The incorporation of this AI tool in creative areas such as architecture is leading to a transformation of the creative process, taking AI not only as tools but as creative collaborators, or as co-creators, because they can only replicate parts of the creative process, and currently cannot recreate the creative process in its entirety (Molina-Siles and Ribera, 2023). The research by Rodríguez et al. (2024) mentions that the use of Midjourney as an educational technological resource generates significant benefits in the teaching-learning processes by enabling the analysis and systematized return of information on the student's progress. This approach favors more accurate and personalized feedback, which contributes to the development of autonomous and adaptive learning, tailored to each student's individual needs. Consequently, this tool promotes the self-management of knowledge, transforming the student into an active, self-taught agent in their own training process.

### 2.3 Academic performance from AI

When analyzing the concept of artificial intelligence and its benefits, we can definitely say that we are referring to a technological tool that can strengthen Higher Education in our country, improve classroom performance among our academics, and facilitate learning among our university students. According to Menacho Ángeles et al. (2024), artificial intelligence poses fundamental challenges that motivate a genuine interest in studying and understanding its benefits for students and teaching practice. Artificial intelligence is an essential tool today for the teaching process, always considering, in the first place, using it responsibly and ethically.

Gutierrez et al. (2023) argues that teachers must follow clear, precise guidelines for the use of artificial intelligence, which should propose rules and principles applicable to all pedagogical activities carried out in the classroom. This technology can be used for a variety of purposes, not only in teaching or research within a university but also in the work environment. It is important to note that artificial intelligence tools are not always suitable for performing all activities in a pedagogical process. Sometimes, they can pose risks to users and to those involved in the teaching-learning process. Therefore, it is very important to be aware of what it implies when referring to ethical issues, the environment, and human rights. Academic performance in technology can vary, so we should not assume that our students will have a genuine interest in using and adapting to artificial intelligence to enhance their school activities. It is relevant to identify in the teaching process that there are several factors, such as economic, cultural, and social, which will be decisive for teachers to successfully design differentiated teaching strategies according to the profiles of our students, and never force the use of new learning procedures using artificial intelligence.

The main aspects we must consider in artificial intelligence are those related to perception, originality, and students' integration in the development of their school activities, as indicated by Peña Miranda et al. (2024). Currently, information circulates rapidly worldwide; it is necessary to use innovative approaches to address the real challenges in academic integrity today, given the potential of artificial intelligence to transform education in our universities. However, it is vitally important to note that there will always be risks associated with the use of artificial intelligence in education. To mention a few: bias, lack of originality in practical work, and plagiarism. Tramallino and Zeni (2024) point out that the success of teachers using artificial intelligence depends to a large extent on their training and preparation in the area. Information security and ethical aspects should always be considered. The important thing is to train students who understand artificial intelligence within the culture they are part of. Therefore, various studies show the need to train our students from an early age. Emphasizing that the use of artificial intelligence acquires greater relevance in our daily lives. For this reason, the current government is considering integrating AI into curricula at the basic educational levels.

### 2.4 AI and education

There is no doubt that, in recent years, the use of technology has increased, improving the educational experience through educational integration on the one hand and better educational planning using artificial intelligence tools, as stated by De La Cruz et al. (2023). Artificial Intelligence in education is developing and integrating more and more into our practice as teachers, enabling us to use it to evaluate our students in a better, more efficient way. In addition, teachers must actively analyze their daily classroom activities, which allows them to transform everyday life and propose new strategies to develop with students. In this way, the teaching work evaluated and developed generates new practices in educational environments that require an ongoing, comprehensive understanding of teacher training improving their pedagogical practices, contributing to their continuous and efficient improvement, and advancing the educational development of their institution.

It is important to mention the relationship between artificial intelligence and university professors today. According to Díaz et al. (2024), an increase in the use of artificial intelligence tools in academic writing has been observed in recent years, although some ethical concerns may arise, especially regarding the autonomy and genuine learning of students when using these tools. However, it is relevant for the development of teaching activities to balance the development of certain skills and, above all, to exercise critical thinking. In this way, we can ensure that there is a real need to reflect on the power that artificial intelligence experts in higher education in our country today. It is a reality that artificial intelligence currently has great potential to transform teaching and learning in higher education in our country. It offers the potential to improve learning efficiency in a highly personalized way for each of our students (González-Geraldo and Ortega-López, 2024). For this reason, it is very important to emphasize that, in recent years, there has been an increase in the use of artificial intelligence in higher education institutions. Among other relevant aspects, intelligent tutorials and data analysis can improve teaching efficiency by training and developing the teaching staff of universities. Therefore, it is important to address the challenges posed by the use and incorporation of artificial intelligence in university teaching staff's performance today.

According to Wilcox et al. (2023), in the context of university education, it is necessary to establish an artificial intelligence system that offers scientific and technological support for new innovative projects, covering the main student demands focused on establishing new work strategies with a teaching team capable of implementing original techniques based on the comprehensive training of university students, solving specific university problems. Romani Pillpe et al. (2025) explain that, based on artificial intelligence, they facilitate teachers‘ tasks, improving their efficiency in planning classes and pedagogical activities and enhancing the teaching-learning process. Currently, more digital tools are being incorporated into the development of teacher activities, with the sole objective of improving students' learning and making it more meaningful. However, using artificial intelligence today poses a significant challenge for our teachers, as well as a challenge for future projections. Its application in the development of its activities represents a new challenge, as it must integrate new technologies to improve its teaching practice in the classroom (Peña et al., 2018). It is important to note that teachers play a role as learning mediators, and that artificial intelligence optimizes educational processes, motivating them to continue increasing their dedication to teaching and to interact directly with students. We must focus on effective integration to achieve this objective. Teachers must develop competencies that allow them to incorporate these new technologies into their pedagogical practices. For Galván-Vela et al. (2024), the integration of artificial intelligence into higher education teaching represents a revolution in how we perceive it and design teaching with our students. In this way, we can understand that artificial intelligence not only offers us tools to automate administrative processes but also enables us to personalize learning, allowing our teachers to adapt their content and improve their pedagogical efficiency.

### 2.5 AI literacy and information literacy

The rapid integration of artificial intelligence-based systems into educational, professional, and social environments has driven the development of new skills related to the critical understanding of technology and the strategic management of information. In this context, two constructs have gained particular relevance in recent scientific literature: AI Literacy and Information Literacy. Both concepts relate to the development of citizens capable of navigating complex digital ecosystems, although each emphasizes specific dimensions.

Yang et al. (2025) mapped research on artificial intelligence literacy in education during the period 2014–2024, using a bibliometric analysis of 335 articles from relevant databases. The findings showed a significant increase in publications starting in 2018, reflecting interdisciplinary growth and the consolidation of key topics such as data literacy. Several others authors agree that AI Literacy encompasses the knowledge, skills, and attitudes necessary to understand, use, critically evaluate, and ethically interact with artificial intelligence systems. In a complementary way, Lintner (2024) conducted a systematic review of AI literacy measurement scales and identified that this construct includes dimensions such as technical understanding, critical thinking, ethics, and responsible use of AI. She points out the need for valid instruments to assess competencies in students and teachers. Sperling et al. (2025) also analyzed how AI literacy is conceptualized in secondary education. They found three approaches: functional (basic use), situated (contextual use), and transformative (social and critical impact). Similarly, Jones (2026) mentions that, in higher education, AI literacy is primarily measured in university students through competencies in use, algorithmic understanding, ethics, and critical thinking. In this sense, authors such as Ali and Richardson (2025) define information literacy as the ability to locate, evaluate, organize, use, and communicate information critically and ethically, indicating that university libraries are integrating AI literacy within information literacy, preparing users to evaluate AI-generated content, and distinguish reliable information. Shahrebabaki (2026) also argues that information literacy in the age of AI no longer only involves searching for information, but also validating automated content, recognizing algorithmic manipulation, and creating knowledge responsibly.

### 2.6 AI and learning

Artificial intelligence, despite its challenges, offers great possibilities for individualizing learning (Ahumada, 2024). Teachers must develop digital skills, but above all, a responsible, serious, reflective attitude toward artificial intelligence. In addition, education must foster a more inclusive environment in universities by providing new opportunities for students to develop their skills and acquire new knowledge that will support their comprehensive education, as well as to integrate skills that help teachers in their teaching-learning process with their students in the twenty-first century. In this way, we can explain that the initial training of teaching staff is at a key moment, motivated by rapid technological progress and the growing needs of a new society in constant evolution. Reflection is an essential component of teachers' professional development. In today's dynamic and panoramic educational system, where technology is increasingly integrated into situated learning and is increasingly transformative in universities, placing a true emphasis on the teacher and his role as a facilitator and guide for his students, creating very meaningful and transcendent learning experiences.

The rapid development of the use and implementation of artificial intelligence is a reality today that offers many opportunities to enhance student development and comprehensive training. However, we are aware of the risks and challenges in the educational area. According to Vicente-Yague-Jara et al. (2023), there are several ways in which intellectual intelligence can be involved in the teaching-learning process today. However, it is important to note that cognitive abilities should not be reduced in this context, and that artificial intelligence cannot replace human intelligence and creativity. It is a reality that artificial intelligence has a lot of information, far more than a person can handle, which is essential to increasing the chances of solving problems by providing greater knowledge of the details in the process of finding possible solutions to school conflicts in the student-teacher relationship.

Galván-Vela et al. (2024) point out that the integration of artificial intelligence in teaching in university higher education with the perception, knowledge of teachers and practical experience will always be fundamental in the process of improvement in the development of our teaching staff, which is why it is relevant to carry out an evaluation of the perception and knowledge of teaching staff about the integration of artificial intelligence in higher education which represents a transcendental factor to be able to understand much better the disposition of teachers toward this tool that represents a competitive advantage in the teaching and learning process in our students today.

### 2.7 AI appropriation index

This study proposes the Artificial Intelligence Appropriation Index as a multidimensional explanatory variable composed of access, frequency of use, digital competence, pedagogical integration, and ethical use. It posits that higher levels of technological appropriation favor university learning and, directly and indirectly, academic performance. The accelerated incorporation of Artificial Intelligence (AI) in higher education has transformed the processes of teaching, learning, assessment, and academic production. Recent studies show that the adoption of AI in universities has gone from being experimental to becoming part of the everyday academic infrastructure, with widespread use among students and faculty (Isaifan, 2026).

However, using AI does not necessarily equate to appropriating it. The literature on educational innovation distinguishes between technological adoption (initial use) and technological appropriation (meaningful, critical, and sustained integration within academic practices). In higher education, this implies that students not only access tools such as conversational assistants, but also develop the capacity to use them strategically, ethically, and for deep learning purposes (Reyes et al., 2025).

**Figure 1:**
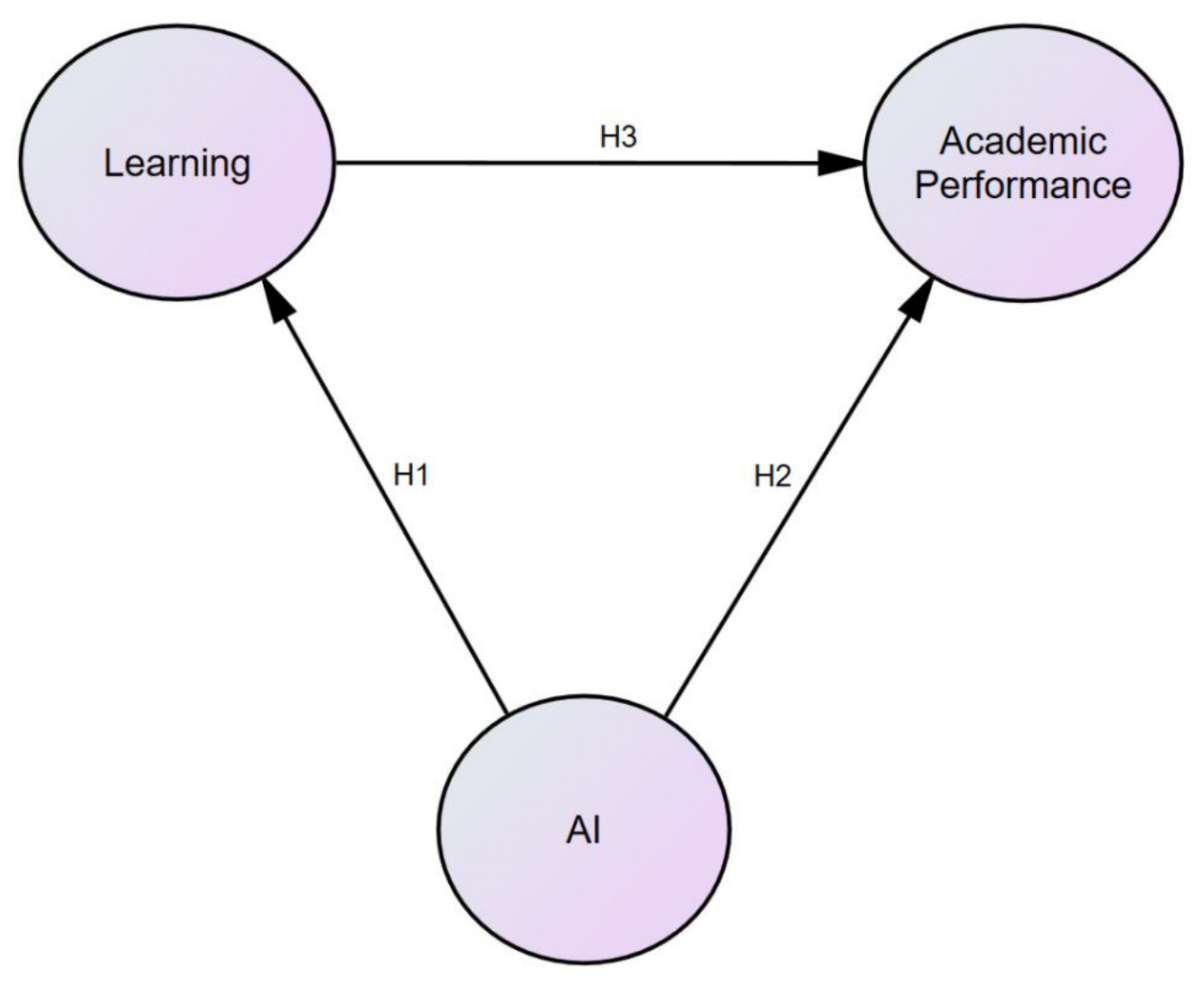
Proposed model.

Based on the model presented in Figure 1, the hypotheses of this research are proposed:

H1: The use of AI in the classroom positively influences the learning of university students

H2: The level of learning of university students is positively and significantly related to their academic performance.

H3: The use of AI in the teaching-learning process positively influences the academic performance of university students.

## 3 Methodology

The research was developed using a quantitative approach and a cross-sectional design, aimed at empirically verifying the proposed hypothesis. To this end, the data were collected and analyzed through the application of the Structural Equation Model (SEM) to examine the particular characteristics of the variables under study, Artificial Intelligence (AI) and Learning, as well as the structural relationships between them. The selection procedure for participants was carried out using simple random sampling, with a universe of 289 students from a university in northwestern Mexico, a margin of error of +/– 5%, and a confidence level of 95% (p, q = 0.5). The instrument was administered to students in the third, fourth, and sixth semesters of the business intelligence career—the business administration career —and to accounting students, respectively, who were randomly selected. The data collection instrument was a structured survey composed of 17 items, organized on a five-point Likert scale. Then, to analyze the statistical processes, the statistical software SPSS version 24 was used. Subsequently the Cronbach's alpha method was applied to verify the internal consistency of the instrument as well as its respective confidence intervals, resulting in a value of 0.938, so the instrument is considered to be highly reliable; see Table 1. The questionnaire was designed based on the three latent variables that comprise the object of study and their respective theoretical dimensions, related to both constructs, thereby guaranteeing the validity and reliability of the instrument applied. Likewise, the scale by Van Auken et al. (2018) was used, consisting of three dimensions: Use of AI Tools, Perception of Usefulness and Effectiveness, and Ethics and Trust in AI. To measure Learning, the scale of Maldonado et al. (2012) is used, comprising three dimensions: Academic Performance, Learning Self-Management, and Motivation and Academic Commitment (see Table 2).

**Table 1:** Instrument reliability.

Cronbach's alpha	Number of items
0.938	17

**Table 2:** Composition of the assessment instrument.

Construct	Dimensions	References	Items	Response type
AI	Use AI tools	Van Auken et al. (2018)	8	5-point Likert scale
	Ethics and trust in AI			
Learning	Learning self-management	Maldonado et al. (2012)	9	
Academic performance	Motivation and academic commitment			

## 4 Results

To ensure the validity of the data obtained, reliability tests were conducted to evaluate the instrument's quality. Such evidence constitutes fundamental references to ensure its relevance in the investigation. In this regard, the scale's internal consistency was examined using Cronbach's alpha, calculated as the average of the item correlations. Likewise, the Composite Reliability Index (CFI) was considered the second measure of the model's robustness, and the Extracted Variance Index (IVE) was incorporated to verify the convergent validity of the variables under study.

Regarding the Artificial Intelligence (AI) construct, the results of the reliability analysis indicate Cronbach's alpha coefficients of 0.832 for the AI Tools dimension, 0.893 for Trust in AI, and 0.891 for Self-Management of Learning. Likewise, the composite reliability (CFI) yielded values of 0.872, 0.893, and 0.891, respectively, indicating adequate internal consistency across all evaluated dimensions. Regarding the Extracted Variance Index (EVI), values of 0.495, 0.671, and 0.623 were obtained, which are considered acceptable within the parameters established in the methodological literature. Taken together, these results confirm that the AI construct demonstrates satisfactory reliability and convergent validity, supporting the statistical robustness of the measurement instruments used.

Regarding the artificial intelligence construct, the results show reliability across the analyzed dimensions. Cronbach's alpha coefficient reached 0.832 for the AI tools dimension and 0.893 for Trust in AI. For the Learning construct, Cronbach's alpha coefficient was 0.891 for the Self-Management of Learning dimension, indicating strong internal consistency. In addition, the Composite Reliability Index (CFI) values were 0.872, 0.893, and 0.891 for each dimension, respectively. Finally, the Extracted Variance Index (IVE) reached values of 0.495, 0.671, and 0.623, corroborating the scales' convergent validity. Taken together, these results indicate that the Artificial Intelligence construct has acceptable reliability.

Regarding the Academic Performance construct, the Cronbach's Alpha for the Motivation and Academic Commitment dimension is 0.842. Regarding the IFC test, the value is 0.842. Similarly, the results for the Extracted Variance Index report a value of 0.687, indicating adequate convergent validity of the model. Consequently, it is concluded that the Academic Performance construct demonstrates satisfactory levels of reliability and internal consistency (see Table 3).

**Table 3:** Data validation.

Construct	Dimensions	(α) Cronbach's alpha	IFC	IVE
IA	AI tools	0.864	0.872	0.495
	Trust in AI	0.893	0.893	0.671
Learning	Learning self-management	0.891	0.891	0.623
Academic performance	Motivation and academic commitment	0.842	0.842	0.687

**Figure 2:**
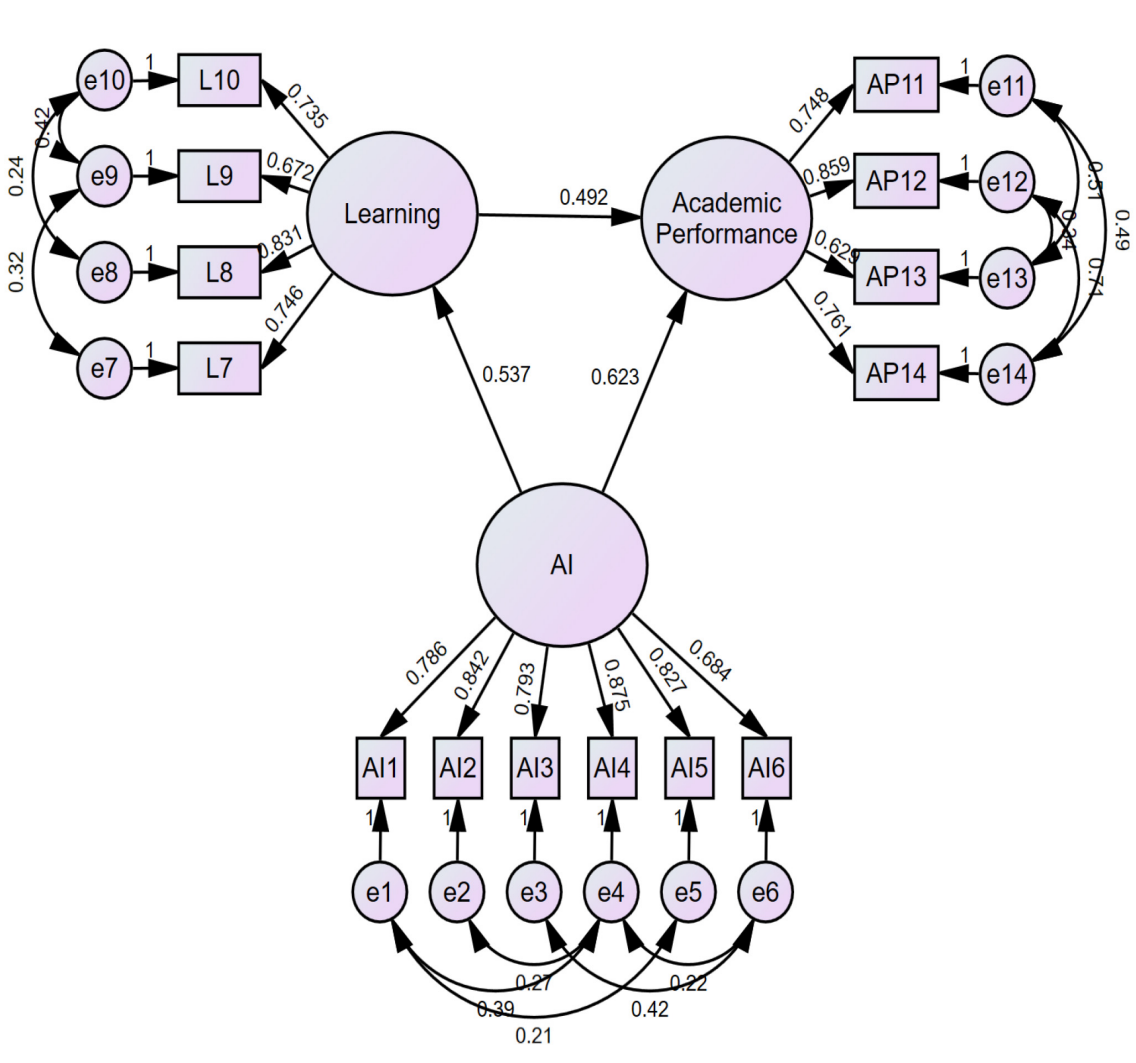
Confirmatory model with added covariances.

In Figure 2 the modified model with covariances shows the factor loadings between the dimensions; values greater than 0.50 in their variances, or very close to this value, are observed, except for item 13 of the Academic performance factor (DA133), which has the lowest WITH of 0.629. Among the 14 factors analyzed, the relationships established by the factor loadings are significant in all cases (*p* = 0.000), with a high covariance (0.62) with the AI factor in Academic Performance.

Table 4 presents the results of the proposed hypotheses, showing the relationships among the latent variables. The t-test was obtained, starting from the fact that values greater than 1.96 indicate that there is a strong relationship between the constructs of the structural model, of which AI → Learning (β =.439, *t* = 4.562, *p* < 0.001); The use of Artificial Intelligence in classroom settings exerts a positive and significant effect on university students' learning outcomes by enhancing academic autonomy, content comprehension, motivation, and engagement. The use of Artificial Intelligence in the classroom has a positive and significant effect on university students' learning outcomes by improving academic autonomy, content comprehension, motivation, and engagement. This indicates that AI significantly contributes to the use of tools that students use to learn from learning outcomes, showing a coefficient of.439, which signifies a positive relationship. The Learning → Academic Performance (β =.781, *t* = 6.845, *p* < 0.001); Learning has a positive and significant effect on university students' academic performance by improving content comprehension, academic autonomy, motivation, and engagement. Enhanced learning outcomes contribute substantially to students' ability to complete academic tasks effectively, achieve higher grades, and improve overall performance indicators. This indicates that learning significantly contributes to academic achievement, showing a coefficient of 0.781, which means a positive relationship. AI → Academic Performance (β =.524, *t* = 5.287, *p* < 0.001) The use of Artificial Intelligence in classroom settings exerts a positive and significant effect on university students' academic performance by enhancing academic autonomy, task efficiency, content comprehension, motivation, and engagement. The use of Artificial Intelligence in the classroom has a positive and significant effect on students' academic outcomes by improving productivity, assignment quality, and overall academic achievement. This indicates that AI significantly contributes to students' academic performance through the effective use of intelligent tools, showing a coefficient of 0.524, which means that there is a high positive and significant relationship between variables.

**Table 4:** Standardized regression coefficient.

Relationship between variables	Route coefficient (β)	Standard deviation (σ)	Statistical *t*	*p*
AI → learning	0.439	0.092	4.562	^***^
Learning → academic performance	0.781	0.028	6.845	^***^
AI → academic performance	0.524	0.065	5.287	^***^

^*^p < 0.05; ^**^p < 0.01; ^***^p < 0.001.

## 5 Discussion

The findings of the present study confirmed the proposed hypotheses regarding the relationships among Artificial Intelligence (AI), learning, and academic performance in higher education. Similar to previous studies on digital transformation in universities, the results indicate that technological tools can generate meaningful improvements in educational processes when they are strategically integrated into academic environments.

Regarding the first hypothesis (H1), the results demonstrated that the use of Artificial Intelligence in classroom settings exerts a positive and significant effect on university students' learning outcomes. This suggests that AI contributes substantially to strengthening academic autonomy, content comprehension, motivation, and student engagement. In particular, the structural coefficient obtained confirms that students perceive AI-based tools as useful resources for organizing information, clarifying concepts, receiving immediate feedback, and supporting self-regulated learning. These findings are consistent with prior literature emphasizing that intelligent educational technologies enhance personalized learning experiences and improve student participation.

The second hypothesis (H2) was also supported, showing that learning has a positive and significant effect on academic performance. This indicates that students who develop stronger comprehension skills, greater motivation, and higher academic involvement tend to obtain better academic results, reflected in task completion, productivity, and overall achievement. Therefore, learning functions as a central explanatory mechanism linking educational innovation with measurable academic success.

Concerning the third hypothesis (H3), the results confirmed that Artificial Intelligence has a positive and significant direct effect on academic performance. This implies that beyond its influence on learning, AI also improves students' efficiency in completing assignments, managing academic workload, and producing higher-quality outputs. Intelligent tools appear to facilitate time management, access to academic resources, and performance optimization, particularly in demanding university contexts. From a methodological perspective, the confirmatory factor analysis validated the multidimensional structure of the proposed constructs. The indicators associated with AI use, learning, and academic performance showed adequate factor loadings, confirming that the measurement items effectively represented their theoretical dimensions. Likewise, the structural equation modeling (SEM) procedure demonstrated satisfactory model fit and statistically significant relationships among latent variables. These results reinforce the importance of properly managing digital technologies within universities. Although AI offers clear benefits for learning and performance, institutions must also address challenges related to digital literacy, ethical use, overdependence, academic integrity, and equitable access. The educational value of AI depends not only on technological availability, but also on pedagogical design, teacher guidance, and responsible student engagement.

In the study by De Jesus et al. (2025), which challenges the use of artificial intelligence in higher education, they mention the limitations of ChatGPT and similar tools in the preparation of academic texts and the development of research activities. It is essential to use them wisely. Although their potential is undeniable, these technologies do not constitute a definitive solution for complex learning processes, especially in academic tasks that demand methodological rigor and conceptual depth. Likewise, an excessive dependence on automation could affect the researcher's ability to strengthen essential competencies, encourage the depersonalization of intellectual work, and promote a reductionist vision of the educational process, focused more on efficiency than on the deep construction of knowledge.

In this sense, the research also identified that AI significantly reduces the time required to deliver high-quality student work, attributed to the use of artificial intelligence tools for automated correction and evaluation. The results show a reduction in time, which directly impacts the learning experience of university students by facilitating the timely identification of errors and the automatic correction of errors, adapting to the needs of each student in the teaching-learning process, thus helping to improve academic performance. For this reason, AI plays a fundamental role in university-level training contexts, where practical skills and competencies are important. However, despite the benefits observed, incorporating AI-based technologies into university teaching poses significant challenges. Based on the importance of restructuring curricular content and adjusting pedagogical strategies according to the capabilities and restrictions of artificial intelligence tools. Finally, the study highlights that AI should not be viewed merely as an auxiliary tool, but as a strategic educational resource capable of transforming learning processes and enhancing academic performance when implemented responsibly. Higher education institutions that proactively adopt AI-oriented pedagogical models may strengthen student success, institutional competitiveness, and future readiness in increasingly digital academic environments.

## 6 Conclusions

Finally, the results highlight the importance of educational institutions continuously evaluating their technology-supported teaching models, establishing clear objectives, defining appropriate pedagogical strategies, and ensuring optimal infrastructure and resources. Likewise, it is essential to prepare both students and teachers for the effective use of AI, fostering advanced digital skills that allow them to fully leverage its potential in the teaching and learning process.

This study confirms that Artificial Intelligence (AI) has become a strategic resource for strengthening teaching–learning processes in higher education. The results of the structural equation model provide empirical evidence that the use and appropriation of AI positively and significantly influence university students' learning outcomes, particularly through improvements in academic autonomy, content comprehension, motivation, and engagement. These findings suggest that AI-based tools can support more dynamic, personalized, and student-centered educational experiences.

Likewise, the study demonstrates that learning exerts a positive and significant effect on academic performance. Students who report stronger learning processes tend to achieve better academic outcomes, reflected in higher productivity, improved task completion, and better overall performance. This confirms the central role of learning as a determinant of student success and as a mechanism through which technological resources generate measurable educational benefits.

In addition, AI was found to have a direct positive effect on academic performance, indicating that intelligent tools not only enhance learning processes but also contribute to efficiency, assignment quality, and academic achievement. Therefore, AI operates through both direct and indirect pathways, with learning acting as a relevant mediating variable in the relationship between AI and performance.

From a practical perspective, these findings highlight that the educational value of AI depends not merely on access to technology, but on its meaningful and responsible appropriation. Universities should therefore move beyond simple technological adoption and promote strategies focused on digital competence, ethical use, pedagogical integration, and critical thinking. Faculty training, curriculum redesign, and institutional policies are essential to maximize the benefits of AI while minimizing risks such as overdependence or superficial learning.

Overall, this research contributes to the emerging literature on AI in higher education by proposing an integrated model linking AI appropriation, learning, and academic performance. The evidence indicates that when implemented strategically, AI can become a transformative factor for improving educational quality and student outcomes in universities.

## Data Availability

The raw data supporting the conclusions of this article will be made available by the authors, without undue reservation.

## References

[B1] Abeliuk A. Gutiérrez C. (2021). History and evaluation of artificial intelligence. Rev. Bits Cienc. 21, 14–21. doi: 10.71904/bits.vi21.2767

[B2] Aguilar G. M. F. Gavilanes D. C. A. Freire E. M. A. Quincha M. L. (2023). Artificial Intelligence and university education: a systematic review. Sci. Mag. Rev. Investig. Innov. 8, 109–131. doi: 10.33262/rmc.v8i1.2935

[B3] Ahumada F. S. (2024). La formación docente en la era digital: práctica reflexiva, aprendizaje situado e inteligencia artificial. Ens. Pedagógicos 19, 4.

[B4] Ali M. Y. Richardson J. (2025). AI literacy guidelines and policies for academic libraries: a scoping review. Educ. Inf. 51. doi: 10.1177/03400352251321192

[B5] Añez Held P. (2023). Generative Artificial Intelligence in the Face of posthuman Disembodiment in the Visual Arts: The Cases DALL-E, Stable Diffusion y Midjourney. Available online at: https://redcol.minciencias.gov.co/Record/UNIANDES2_4e163569ad3fd2303f83521512d59a2a/Details (Accessed March 18, 2026).

[B6] Bond M. Bedenlier S. Marín V. I. Händel M. (2024). Artificial intelligence in higher education: a systematic review of emerging research and applications. Comput. Educ. Artif. Intell. 5:100168. doi: 10.1186/s41239-023-00436-z

[B7] Briva-Iglesias V. (2023). Artificial Intelligence (AI). Textual Learning Resource. Barcelona: Fundació Universitat Oberta de Catalunya (FUOC).

[B8] Campo-Ruiz I. (2025). Artificial intelligence may affect diversity: architecture and cultural context reflected through ChatGPT, Midjourney, and Google Maps. Humanit. Soc. Sci. Commun. 12, 24. doi: 10.1057/s41599-024-03968-5

[B9] Coeckelbergh M. (2021). É*tica de la Inteligencia Artificial*. Madrid: Comercial Grupo ANAYA, SA. Spanish.

[B10] Corredera J. C. (2023). Generative artificial intelligence. An. Real Acad. Doct. 8, 475–489.

[B11] Corzo P. (2025). Urban visual culture through generative artificial intelligence: spectacle and beautification in the city of Córdoba, Argentina. Comun. Soc. 1–21. doi: 10.32870/cys.v2025.8818

[B12] Cotton D. R. E. Cotton P. A. Shipway J. R. (2024). Chatting and cheating? Ensuring academic integrity in the era of ChatGPT. Innov. Educ. Teach. Int. 61, 228–239. doi: 10.1080/14703297.2023.2190148 (Accessed February 18, 2026).

[B13] De Jesús Molina T. Infante Miranda M. E. Ruiz Quiroz J. F. Burbano García L. H. (2025). Challenges of using Artificial Intelligence in higher Education. Libr. An. Investig. 21, 1–14. Available online at: https://revistasbnjm.sld.cu/index.php/BAI/article/view/1054

[B14] De Jesus L. S. Santos C. R. Caires N. O. (2025). Gestão de pessoas na era da inteligência artificial e automação. Rev. Multidiscip. Nord. Mineiro 9, 1–10.

[B15] De La Cruz M. A. T. Benites E. M. M. Cachinelli C. G. C. Caicedo E. V. A. (2023). Incidencias de la inteligencia artificial en la educación. Recimundo 7, 238–251. doi: 10.26820/recimundo/7.(2).jun.2023.238-251

[B16] Díaz J. P. R. Méndez C. D. L. M. C. Nieves Z. J. L. (2024). Uso de modelos de inteligencia artificial en la optimización de la enseñanza de matemáticas en la educación superior. Reincisol. 3, 4334–4355. doi: 10.59282/reincisol.V3(6)4334-4355

[B17] Díaz-Ramírez J. (2021). Machine Learning and deep Learning. Ingeniería. 29, 180–181. doi: 10.4067/S0718-33052021000200180

[B19] Dumancela C. A. B. Viteri B. S. S. Rea D. W. G. Lema B. E. C. (2024). Application of artificial intelligence in solving mathematical and statistical problems. Reincisol. 3, 3117–3145. doi: 10.59282/reincisol.V3(6)3117-3145

[B20] Dwivedi Y. K. Kshetri N. Hughes L. Slade E. L. Jeyaraj A. Kar A. K. . (2023). So what if ChatGPT wrote it? Multidisciplinary Perspectives on Opportunities, Challenges and Implications of generative conversational AI for Research, Practice and Policy. Int. J. Inf. Manage. 71:102642. doi: 10.1016/j.ijinfomgt.2023.102642

[B21] Franganillo J. (2023). Generative Artificial Intelligence and its Impact on the Creation of media Content. Methaodos Rev. Cienc. Soc. 11:m231102a10. doi: 10.17502/mrcs.v11i2.710

[B22] Galván-Vela E. Ripoll R. R. Altamirano M. A. S. Rodriguez D. M. S. (2024). El trinomio compromiso, satisfacción y justicia organizacional en el binomio felicidad e intención de rotar. Retos 14, 187–202.

[B23] González-Geraldo J. L. Ortega-López L. (2024). Can AI fool us? University students' lack of ability to detect ChatGPT [¿Puede engañarnos una IA? Carencias del estudiantado universitario para detectar ChatGPT]. Educ. Knowl. Soc. 25:e31760.

[B24] González-Videgaray M. Romero-Ruiz R. (2022). Artificial intelligence in education: from passive users to critical creators. Figuras Rev. Acad. Investig. 4, 48–58. doi: 10.22201/fesa.26832917e.2022.4.1.243

[B25] Gutierrez C. I. Aguirre A. Uuk R. Boine C. C. Franklin M. (2023). A proposal for a definition of general purpose artificial intelligence systems. Digit. Soc. 2, 36. doi: 10.1007/s44206-023-00068-w

[B26] Holmes W. Bialik M. Fadel C. (2022). Artificial Intelligence in Education: Promises and Implications for Teaching and Learning. Boston, MA: Center for Curriculum Redesign.

[B27] Isaifan R. J. (2026). Artificial intelligence in higher education: a global statistical synthesis for policy and quality assurance reform. Educ. Sci. 16:483. doi: 10.3390/educsci16030483

[B28] Jones J. (2026). On the measurement of AI literacy among students in higher education: a scoping review. Int. J. AI Pedagog. Innov. Learn. Futures. 1. doi: 10.46787/ijaipil.v2026i1.6920

[B29] Kasneci E. Sessler K. Küchemann S. Bannert M. Dementieva D. Fischer F. . (2023). ChatGPT for good? On opportunities and challenges of large language models for education. Learn. Individ. Differ. 103:102274. doi: 10.1016/j.lindif.2023.102274

[B30] Lee K. F. (2020). Superpotencias de la Inteligencia Artificial. Madrid: Grupo Planeta. Spanish.

[B31] Lintner T. (2024). A systematic review of AI literacy scales. npj Sci. Learn. 9:50. doi: 10.1038/s41539-024-00264-4 39107327 PMC11303566

[B32] López Baroni M. J. (2019). The narratives of artificial intelligence. Rev. Bioét. Derecho. 46, 5–28.

[B33] Luckin R. Holmes W. Griffiths M. Forcier L. B. (2022). Intelligence Unleashed: An Argument for AI in Education. London: Pearson Education.

[B34] Maldonado G. Sánchez J. Gaytán J. García R. (2012). Measuring the Competitiveness Level in Furniture SMEs of Spain. Int. J. Econ. Manage. Sci. 1, 9–19.

[B35] Menacho Ángeles M. R. Pizarro Arancibia L. M. Osorio Menacho J. A. Osorio Menacho J. A. León Pizarro B. L. (2024). Inteligencia artificial como herramienta en el aprendizaje autónomo de los estudiantes de educación superior. Rev. INVECOM 4. doi: 10.5281/zenodo.10693945

[B36] Molina-Siles P. Ribera M. G. (2023). Artificial intelligence and creativity for the generation of architectural images from textual descriptions in midjourney. emulating Louis I. Kahn. EGA Expresión Gráfica Arquitectónica. 28, 238–251. doi: 10.4995/ega.2023.19294

[B37] Nogueira G. F. (2019). Educação básica e inteligencia artificial: perspectivas, contribuições e desafios [Tesis de mestrado em Educação]. Pontifícia Universidade católica de São Paulo. Available online at: https://tede2.pucsp.br/bitstream/handle/22788/2/Francielle%20Nogueira%20Gatti.pdf

[B38] Peña Miranda S. Bueno Doral T. García Castillo N. (2024). Ética y discurso en la inteligencia artificial periodística: análisis de contenido de noticias sobre migración en España generadas por ChatGPT. *Sphera Publica* 2.

[B39] Peña V. R. G. Marcillo A. B. M. Ramírez J. A. Á. (2018). Artificial intelligence in education. Knowl. Hub Rev. Cient.-Prof. 3, 214–231.

[B40] Peña V. R. G. Marcillo A. B. M. Ramírez J. A. Á. (2020). La inteligencia artificial en la educación. Dominio de las Cienc. 6, 28.

[B41] Reyes G. Tolozano-Benites R. George-Reyes C. Rumbaut D. Barzola-Monteses J. (2025). Adoption of artificial intelligence applications in higher education. Front. Educ. 10:1680401. doi: 10.3389/feduc.2025.1680401

[B42] Rodríguez C. M. Zepeda M. G. L. Jaimes K. G. (2024). Use and application of AI as a creative strategy in teaching and teaching practice in the creative industries, midjourney; illustration and photography. Rev. Investig. Latinoam. Competitividad Organizacional. 6, 12–30. doi: 10.51896/rilco.v6i23.642

[B43] Romani Pillpe G. Macedo Inca K. S. Soto Loza G. E. Franco Guevara A. M. Ore Choque M. K. (2025). Revisión sistemática de inteligencia artificial generativa (GenIA) para el diseño de experiencias de aprendizaje, 2020–2025. Rev. Esp. 46, 13–27. doi: 10.48082/espacios-a25v46n03p02

[B44] Rouhiainen L. (2018). Inteligencia Artificial. Madrid: Alienta Editorial. Spanish.

[B45] Ruiz K. K. (2023). Periodic Table of Anderson and Krathwohl's Taxonomy with Artificial Intelligence Apps. Available online at: https://bit.ly/3LAWSIH?r=qr (Accessed March, 2026).

[B46] Sabzalieva E. Valentini A. (2023). ChatGPT and Artificial Intelligence in Higher Education. Paris: UNESCO. Available online at: https://unesdoc.unesco.org/ark:/48223/pf0000385146_spa/PDF/385146spa.pdf.multi (Accessed April, 2026).

[B47] Sánchez F. J. J. (2023). La Inteligencia Artificial Tex-to-Image en el Aula. Mayéutica 2.0. Projects in Arts and Humanities, 158. Available online at: https://uhra.herts.ac.uk/id/eprint/14480/1/tlpah.pdf#page=163 (Accessed March, 2026). doi: 10.58909/ad23869102

[B48] Shahrebabaki M. M. (2026). Information Literacy in the Age of Artificial Intelligence. Berlin: Springer.

[B49] Sperling K. Stenliden L. Mannila L. Hallström J. Nordlöf C. Heintz F. (2025). Perspectives on AI Literacy in middle school classrooms: an integrative review. Postdigital Sci. Educ. 7, 719–749. doi: 10.1007/s42438-025-00560-1

[B50] Tafur A. T. V. Molina R. E. F. (2023). Impact of artificial intelligence on education. Educatio Siglo XXI. 41, 235–264. doi: 10.6018/educatio.555681

[B51] Tramallino C. P. Zeni A. M. (2024). Avances y discusiones sobre el uso de inteligencia artificial (IA) en educación. Educación 33, 29–54. doi: 10.18800/educacion.202401.M002

[B52] Vaerenbergh S. V. (2024). Artificial Intelligence to enhance Creativity and educational Innovation. Available online at: https://dehesa.unex.es/server/api/core/bitstreams/b8b60e86-9c62-4eaf-b1f3-024dd8cfa723/content (Accessed March, 2026).

[B53] Van Auken H. Madrid A. García D. (2018). Innovation and performance in spanish manufacturing SMEs. Int. J. Entrep. Innov. Manage. 8, 36–56.

[B54] Vicente-Yague-Jara M. I. López-Martínez O. Navarro-Navarro V. Cuéllar-Santiago F. (2023). Writing, creativity, and artificial intelligence. ChatGPT in the university context. Commun. Rev. Cient. Comun. Educ. 31, 47–57. doi: 10.3916/C77-2023-04

[B55] Wilcox E. G. Pimentel T. Meister C. Cotterell R. Levy R. P. (2023). Testing the predictions of surprisal theory in 11 languages. Trans. Assoc. Comput. Linguist. 11, 1451–1470. doi: 10.1162/tacl_a_00612

[B56] Yang K. Cai J. Ouyang L. Vasluianu F. A. Timofte R. Ding J. . (2025). “TIRE 2025 challenge on single image reflection removal in the wild: Datasets, methods and results,” in Proceedings of the Computer Vision and Pattern Recognition Conference, 1301–1311.

[B57] Zawacki-Richter O. Marín V. I. Bond M. Gouverneur F. (2019). Systematic review of research on artificial intelligence applications in higher education. Int. J. Educ. Technol. High. Educ. 16:39. doi: 10.1186/s41239-019-0171-0

